# Modulation of Erythrocyte Plasma Membrane Redox System Activity by Curcumin

**DOI:** 10.1155/2016/6025245

**Published:** 2016-01-19

**Authors:** Prabhakar Singh, Rajesh Kumar Kesharwani, Krishna Misra, Syed Ibrahim Rizvi

**Affiliations:** ^1^Department of Biochemistry, University of Allahabad, Allahabad 211002, India; ^2^Division of Applied Science & Indo-Russian Center For Biotechnology (IRCB), Indian Institute of Information Technology, Allahabad 211012, India

## Abstract

Plasma membrane redox system (PMRS) is an electron transport chain system ubiquitously present throughout all cell types. It transfers electron from intracellular substrates to extracellular acceptors for regulation of redox status. Curcumin, isolated from* Curcuma longa,* has modulatory effects on cellular physiology due to its membrane interaction ability and antioxidant potential. The present study investigates the effect of curcumin on PMRS activity of erythrocytes isolated from Wistar rats* in vitro* and* in vivo* and validated through an* in silico* docking simulation study using Molegro Virtual Docker (MVD). Effects of curcumin were also evaluated on level of glutathione (GSH) and the oxidant potential of plasma measured in terms of plasma ferric equivalent oxidative potentials (PFEOP). Results show that curcumin significantly (*p* < 0.01) downregulated the PMRS activity in a dose-dependent manner. Molecular docking results suggest that curcumin interacts with amino acids at the active site cavity of cytochrome *b*_5_ reductase, a key constituent of PMRS. Curcumin also increased the GSH level in erythrocytes and plasma while simultaneously decreasing the oxidant potential (PFEOP) of plasma. Altered PMRS activity and redox status are associated with the pathophysiology of several health complications including aging and diabetes; hence, the above finding may explain part of the role of curcumin in health beneficial effects.

## 1. Introduction

Plasma membrane redox system (PMRS) is an electron transport chain system ubiquitously present in all cell types that transfers electrons from intracellular substrates to extracellular acceptors maintaining redox homeostasis for a successful cell physiology [1]. PMRS has been suggested to play a vital role in reducing oxidative stress; this property has been hypothesized to control the rate of aging, lifespan, and many pathological conditions associated with increased oxidative stress [2, 3]. Proposed functions of PMRS include maintenance of redox state in proteins, stimulation of cell growth, reduction of lipid hydroperoxides, recycling of *α*-tocopherol, reduction of ferric ion prior to iron uptake by a transferring-independent pathway, and the maintenance of the extracellular concentration of ascorbic acid [2–6]. In addition, PMRS also regulates physiological functions like cell metabolism, cellular growth, activity of ion channels, and cell death against changes in redox potential [7, 8]. PMRS activity also plays an important role affecting recycling of extracellular ascorbic acid, thus preventing its depletion [9]. PMRS donates electrons to extracellular ascorbate free radical (AFR) derived from intracellular redox molecules like glutathione (GSH), L-ascorbic acid, nicotinamide adenine dinucleotide (NADH), and other reduced equivalents [9, 10].

Cellular physiology is modulated by oxidative stress mediated changes in redox status specially reduced GSH [11]. GSH is a hydrophilic antioxidant with nucleophilic thiol group and has been reported to participate in regulation of gene expression, protein synthesis, cell proliferation, signal transduction, cytokine production, apoptosis, immune response, and protein glutathionylation [11]. GSH protects the cell and biomolecules against oxidative injury, thus providing a powerful antioxidant defense mechanism against reactive oxygen species (ROS) and reactive nitrogen species (RNS) [12–14].

Curcumin ((1*E*,6*E*)-1,7-bis(4-hydroxy-3-methoxyphenyl)-1,6-heptadiene-3,5-dione), a biphenolic molecule isolated from turmeric (*Curcuma longa*) has pleotropic health protective effects including antioxidation, anti-inflammation, cardiovascular protection, and anticancerous and antiangiogenic properties [15, 16]. The broad range of curcumin's biological properties is due to its ability to bind to over 100 identified different molecular targets [17]. Curcumin modulates cellular activity through interacting with membrane-bound proteins and modulating signaling cascade activity by altering the fluidity of the membrane bilayer [17]. The role of curcumin in regulating redox status is complicated and is not still fully understood. The present study was conducted to investigate the* in vitro* dose-dependent effect of curcumin on PMRS activity of erythrocytes and validated through* in vivo* studies on Wistar rats. We also report the* in silico* docking of curcumin with cytochrome *b*_5_ reductase comparing MoleDock and H-bond score with natural ligand *β*-NADH. The effects of curcumin were also evaluated on redox status in plasma as well as erythrocytes in terms of GSH concentration and plasma ferric equivalent oxidative potentials (PFEOP).

## 2. Material and Methods

### 2.1. Chemicals and Instrument

Curcumin was purchased from Bio Basic Inc., Ontario, Canada (cat. number* #*CB0346). DMPD (*N*,*N*-dimethyl-*p*-phenylenediamine dihydrochloride) was purchased from Sigma Aldrich, India. Other chemicals of highest purity were purchased from Merck, India, and HIMEDIA Labs, India. Digital pH meter Cyber scan PH 500 manufactured by Merck was used to measure the pH of solutions. Spectrophotometric measurements were performed on UV-VIS Spectrophotometer (Shimadzu-UV-1800, Japan).

### 2.2. Experimental Study

#### 2.2.1. Animals

Male albino rats (Wistar strain) weighing between 150 and 200 g were purchased from CDRI, Lucknow, India. Animals were housed in polypropylene cages (6 rats per cage) at 24 ± 2°C under 12 h light: 12 h dark cycles. Animals were fed with standard pellet diet obtained from Dayal Industries Limited, Lucknow, India, and had free access to drinking water. The protocol of the study was in conformity with the guidelines of the Institutional Ethical Committee, University of Allahabad. Twenty-four rats were randomly divided into four groups (six rats/group): Group (I): control, receiving no treatment/supplementation; Group (II): experimental control, rats that were supplemented with 0.9% NaCl solution through oral route; Group (III): curcumin treated group (340 mg/kg b.w., saline); Group (IV): curcumin treated group (170 mg/kg b.w., saline). Oral supplementation of curcumin and sacrifices of rats were performed according to the method of Singh and Rizvi [18].

#### 2.2.2. Preparation of Packed Red Blood Cells and* In Vitro* Experiments with Curcumin

The heparinized blood of rats was centrifuged at 800 ×g at 4°C for 10 min. Fresh plasma was used for experimental analyses. After the removal of plasma, buffy coat, and upper 15% of the packed cells, remaining PRBCs were washed twice with 10 mM phosphate buffer saline pH 7.4.* In vitro* effects of curcumin were observed by incubating washed erythrocytes with curcumin (10^−5^ M to 10^−8^ M) for 30 min at 37°C as described previously [19].

#### 2.2.3. Measurement of PMRS Activity

The PMRS activity was measured following the method of Avron and Shavit [20]. Briefly 0.2 mL of PRBCs was suspended in phosphate buffer saline (PBS) containing 5 mM glucose and 1 mM potassium ferricyanide (fresh) to a final volume of 2.0 mL. The suspensions were incubated for 30 min at 37°C and then centrifuged at 1000 ×g at 4°C for 5 min. The collected supernatant was assayed for ferrocyanide content using 4,7-diphenyl-1,10-phenanthroline disulfonic acid disodium salt and measuring absorption at 535 nm. The activity of PMRS was calculated by using extinction coefficient, *ℰ* = 20,500 M^−1^ cm^−1^. PMRS activity was expressed in *μ*mol ferrocyanide/mL PRBC/30 min.

#### 2.2.4. Measurement of Glutathione

Plasma or erythrocyte GSH was measured according to the method of Beutler [21]. The method is based on the ability of the –SH group of GSH to reduce 5,5′-dithiobis,2-nitrobenzoic acid (DTNB) into a yellow colored anionic product whose absorbance was measured at 412 nm. An aqueous solution of known reduced glutathione concentration was used for standard calibration. Concentration of GSH was determined by using standard plot. Concentration of GSH is expressed in *μ*g/mL plasma and mg/mL PRBCs.

#### 2.2.5. Oxidant Potential of Plasma

The plasma oxidant capacity in terms of plasma ferric equivalent oxidative potential (PFEOP) was measured according to method of Mehdi and Rizvi [22]. Briefly 100 mM stock solution of DMPD was prepared by dissolving 209 mg of DMPD in 10 mL of distilled water. 100 mM acetate buffer (pH 5.25) was prepared by mixing of 100 mM sodium acetate (75.98 mL) and 100 mM acetic acid (24.02 mL) to bring the pH 5.25. Now 1 mL of DMPD stock solution was added to 100 mL acetate buffer (0.1 M, pH 5.25) to bring DMPD concentration to 1 mM. A standard curve of ferric chloride was prepared by taking 0.020–0.20 mM ferric chloride as final concentration in 2 mL solutions (1.9 mL DMPD solution with 100 *μ*L ferric chloride solutions accordingly). Final solutions were kept for 15 minutes and absorbance was taken at 505 nm against a DMPD blank. 100 *μ*L (4 times) of diluted plasma was added to the 1.90 mL of DMPD working solution (1 mM in acetate buffer). The whole content was mixed well and kept for incubation for 10 minutes at room temperature and then centrifuged for 5 minutes at 6000 ×g at 4°C. Absorbance of supernatant was taken at 505 nm against DMPD solution devoid of plasma as blank. The finding absorbance was compared to ferric iron standard curve for final concentrations. Plasma oxidant capacity is reported as mM ferric equivalent/L of plasma.

### 2.3. Computational Study

#### 2.3.1. Protein and Ligands Structure Preparation

Human NADH-cytochrome *b*_5_ reductase with 3-dimensional structure was downloaded from the protein data bank (pdb id = 1 umk, resolution = 1.75 Å) [23, 24]. Input file of protein was prepared by using Molegro Virtual Docker (MVD). The missing hydrogen atom and bond order information were assigned. The active site cavity of target protein was selected based on Kesharwani et al. [25]. The structure of curcumin and other selected ligands were downloaded from pubchem database and prepared by using MVD.

#### 2.3.2. Docking Simulation

Docking simulation study was performed by Molegro Virtual Docker (MVD), an automated docking software on HP Z800 workstation. The simulation results returned five different poses with their MoleDock and H-bond score [26].

### 2.4. Statistical Analysis

Statistical analysis was performed by Graph Pad Prism 5 version 5.01 (Graphpad Software Inc., San Diego, California, USA). One way analysis of variance (ANOVA) was done to assess relationships between parameters followed by Bonferroni's multiple comparison test for comparisons between the various treated groups. All the values with *p* < 0.05 were considered as statistically significant.

## 3. Results

**Figure 1:**
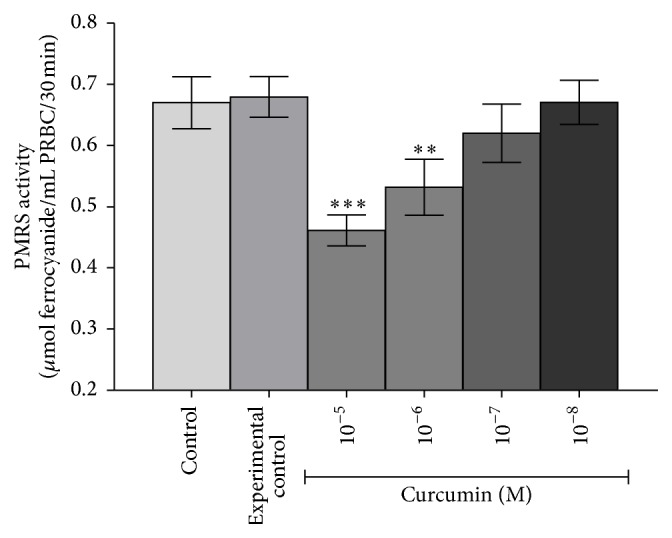
*In vitro* effects of curcumin (10^−5 ^M to 10^−8 ^M) on Wistar rat erythrocytes PMRS activity: PMRS activity expressed in terms of *μ*mol ferrocyanide/mL PRBC/30 min. Values represent mean ± SD. Control represents erythrocytes receiving no treatment; Experimental control: erythrocytes were incubated with solvent, that is, 0.1% DMSO.

**Figure 2:**
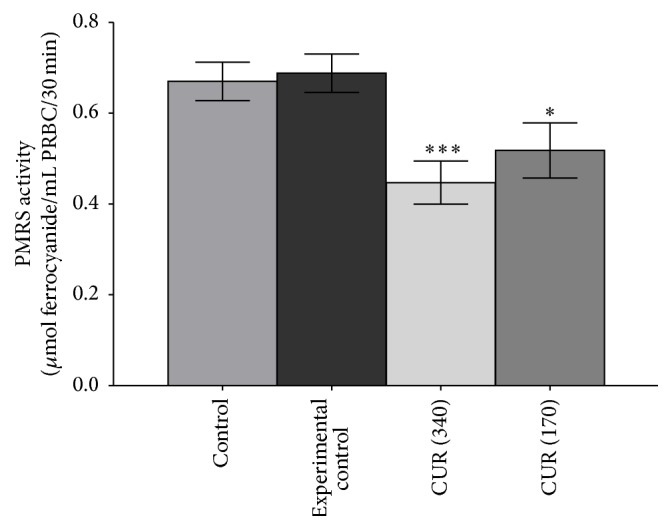
*In vivo* effect of curcumin (340 mg/kg b.w. as CUR 340 and 170 mg/kg b.w. oral CUR 170) on healthy rat erythrocytes membrane PMRS activity. PMRS activity expressed in terms of *μ*mol ferrocyanide/mL PRBC/30 min. Values represent mean ± SD. Control represents rats receiving no treatment/supplementation; experimental control: rats were supplemented with 0.9% NaCl solution through oral route.

**Figure 3:**
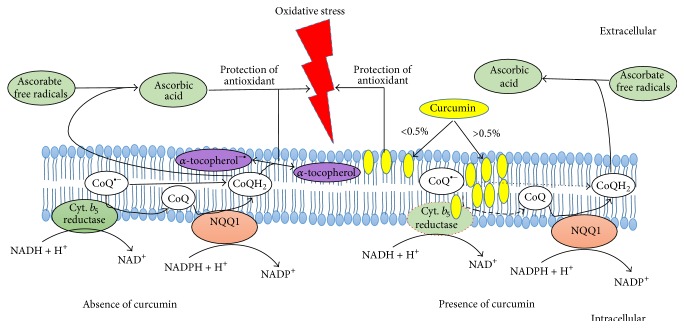
Proposed action mechanism of curcumin: dotted outline of PMRS component and arrow indicates the active targets of curcumin towards downregulation of the PMRS activity.

Figure 1 shows that* in vitro* curcumin (10^−5^ M to 10^−8^ M) triggered concentration dependent downregulation of PMRS activity in erythrocytes obtained from Wistar rats. Maximum downregulation of PMRS activity was observed at 10^−5^ M (*p* < 0.001) of curcumin; decreasing effect was observed on lowering the concentration of curcumin till 10^−8^ M.* In vivo* oral supplementation of curcumin (340 and 170 mg/kg b.w.) to rats significantly (*p* < 0.001) reduced the PMRS activity of erythrocytes as compared with control and experimental control rats (Figure 2). A less pronounced effect was observed at lower concentrations of curcumin; the effect was similar to* in vitro* observations.  Figure 3 shows a proposed action mechanism of curcumin for PMRS activity.

**Figure 4:**
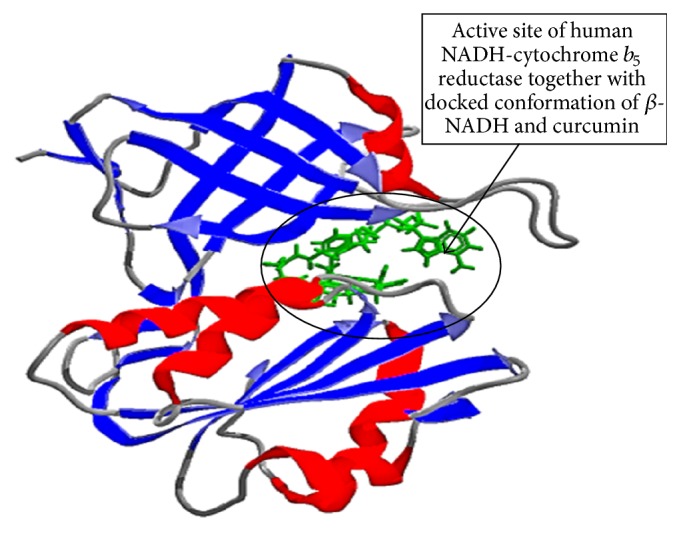
Secondary structure (cartoon) representation at the active site of human NADH-cytochrome *b*_5_ reductase together with docked conformation of ligand *β*-NADH and curcumin.

**Figure 5:**
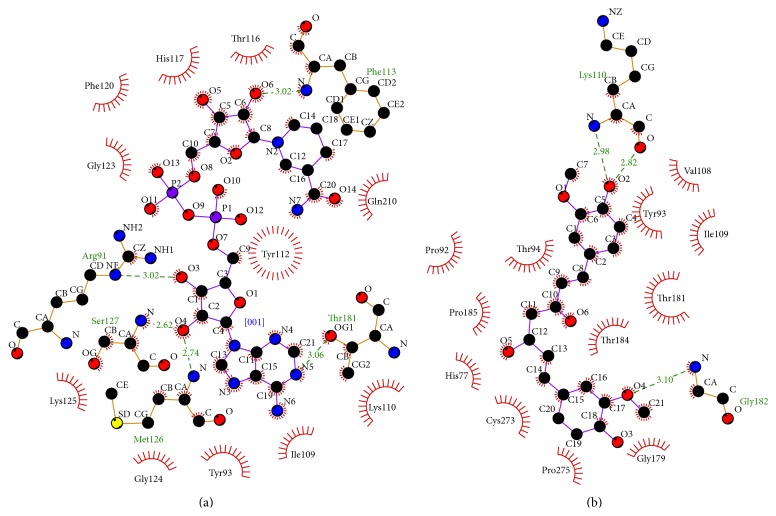
(a, b) Docked conformation of hydrogen bonding view and hydrophobic interaction of (a) *β*-NADH and (b) curcumin with amino acids of human NADH-cytochrome *b*_5_ reductase protein (PDB: 1 umk.pdb) at the active site cavity (hydrogen bonds as green dashed lines between the atoms involved and hydrophobic contacts as an arc with spokes radiating towards the ligand atoms).

The secondary structure (cartoon) representation of active site of human NADH-cytochrome *b*_5_ reductase (1 umk.pdb) together with docked conformation of ligands is shown in Figure 4. Molecular docking simulation results depicted in Figure 5(a) and Table 1 show the active site residues of catalytic unit of human NADH-cytochrome *b*_5_ reductase (1 umk.pdb).* Arg91*, Tyr93, Ile109, Lys110, Tyr112,* Phe113*, Thr116, His117, Phe120, Gly123, Gly124, Lys125,* Met126*,* Ser127*,* Thr181,* and Gln210 were actively involved in hydrogen bonding and hydrophobic interaction with *β*-NADH.  Figure 5(b) shows that curcumin interacted actively with His77, Pro92, Tyr93, Thr94, Val108, Ile109,* Lys110*, Gly179, Thr181,* Gly182*, Thr184, Pro185, Cys273, and Pro275 amino acids at the catalytic center. Five and two hydrogen bonds were formed with amino acids present at the active site by *β*-NADH and curcumin, respectively.  Table 2 shows that the MoleDock score and H-binding energy of curcumin were relatively lower when compared with natural ligand of NADH-cytochrome *b*_5_ reductase (*β*-NADH).

**Figure 6:**
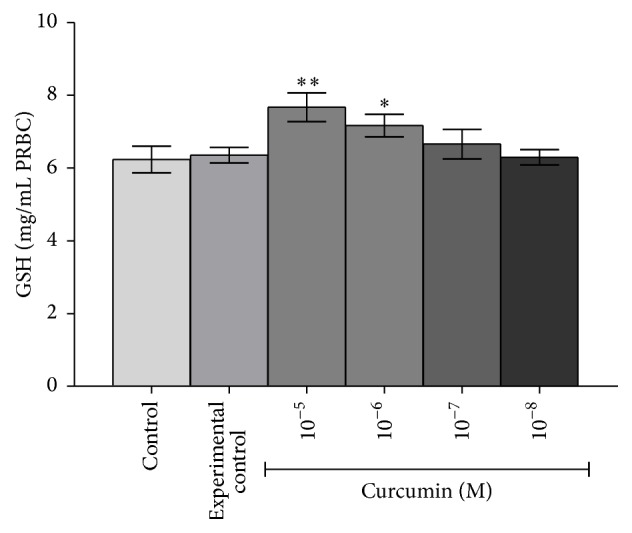
*In vitro* effects of curcumin (10^−5 ^M to 10^−8 ^M) on GSH concentration in Wistar rat RBCs. Concentration of GSH is expressed in mg/mL PRBCs. Values represent mean ± SD. Control represents erythrocytes receiving no treatment; experimental control: erythrocytes were incubated with solvent, that is, 0.1% DMSO.

**Figure 7:**
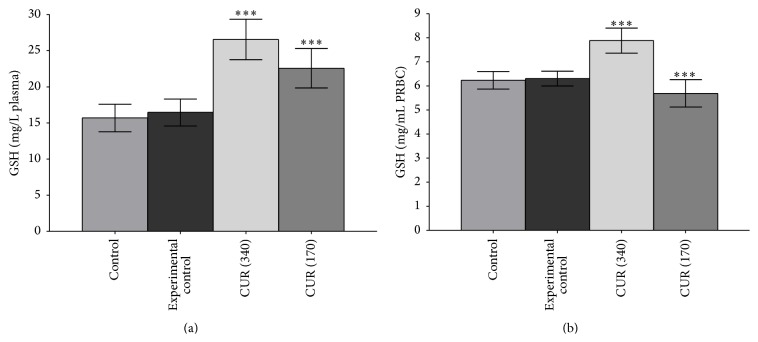
*In vivo* effect of curcumin (340 mg/kg b.w. as CUR 340 and 170 mg/kg b.w. oral CUR 170) on GSH concentration in rats (a) plasma and (b) erythrocytes. Concentration of GSH is expressed in *μ*g/mL plasma and mg/mL PRBCs. Values represent mean ± SD. Control represents rats receiving no treatment/supplementation; experimental control: rats were supplemented with 0.9% NaCl solution through oral route.

**Figure 8:**
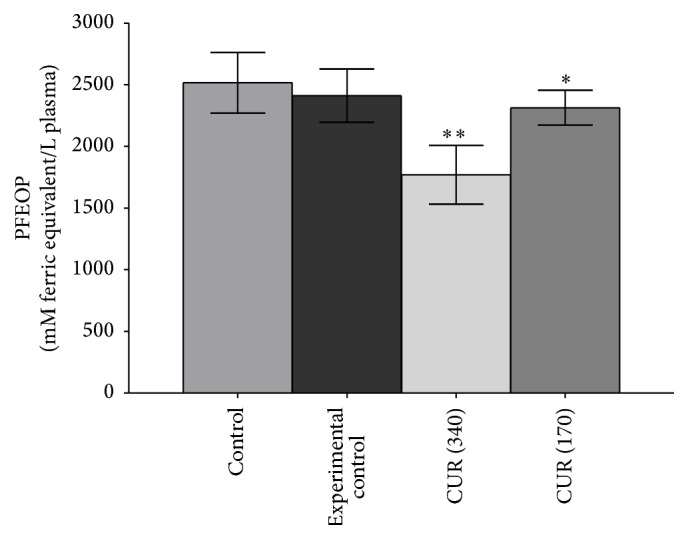
*In vivo* effect of curcumin (340 mg/kg b.w. as CUR 340 and 170 mg/kg b.w. oral CUR 170) on plasma oxidant potential of rat subjected. Plasma oxidant potential is expressed in mM ferric equivalent/L plasma. Values represent mean ± SD. Control represents rats receiving no treatment/supplementation; experimental control: rats were supplemented with 0.9% NaCl solution through oral route.

Figure 6 shows that,* in vitro*, incubation of curcumin (10^−5^ M to 10^−8^ M) with rat erythrocytes significantly (*p* < 0.001) increased the GSH concentration; the maximum effect was observed at 10^−5^ M of curcumin; lower concentrations elicited lesser response.* In vivo,* curcumin (340 and 170 mg/kg b.w.) supplementation significantly (*p* < 0.001) increased GSH concentration in plasma as well as erythrocytes (Figures 7(a) and 7(b)).  Figure 8 shows that* in vivo* curcumin (340 and 170 mg/kg b.w.) supplementation significantly (*p* < 0.001) reduced the oxidant potential of plasma (PFEOP).

## 4. Discussion

The erythrocyte PMRS plays an important role in regulating antioxidant status of the plasma during aging and progression of age associated diseases [27, 28]. Rizvi et al. proposed that long living species have inherent higher PMRS activity in erythrocyte which provides an effective armament to combat oxidative stress [2]. Dietary polyphenols have modulatory effects on PMRS activity which is linked to their cellular internalization [3]. Curcumin has been reported to interact with phospholipid head groups in the membrane bilayer through hydrogen bonds at low concentrations and hydrophobic tail region at high concentrations; these interactions may induce changes in membrane fluidity and associated transport system activity [29, 30].

Our results show that curcumin downregulate PMRS activity in healthy erythrocytes* in vitro* (Figure 1) as well as* in vivo* (Figure 2) in a dose-dependent manner. The observed effect might be due to the biphasic interaction of curcumin with membrane bilayer and/or interacting with amino acids at the active site of PMRS. A hypothetical proposed molecular mechanism of curcumin on PMRS activity is depicted in given Figure 3.

PMRS consists of oxidoreductases and electron carrier protein as major entities in which cytochrome *b*_5_ reductase (EC 1.6.2.2) is a key enzyme. The proposed mechanism elucidates that interaction of curcumin with head groups of membrane can mitigate oxidative stress outside of membrane without influencing PMRS activity. However, interaction at hydrophobic tail region of membrane at higher concentration of curcumin reduces the activity of PMRS.

Figure 4, shows the active site of human NADH-cytochrome *b*_5_ reductase (1 umk.pdb). Results of molecular docking simulation suggest that curcumin actively interacted to form hydrogen bond with amino acids:* Lys110* and* Gly182*, and hydrophobic interaction with His77, Pro92, Tyr93, Thr94, Val108, Ile109, Gly179, Thr181, Thr184, Pro185, Cys273, and Pro275 amino acids at the active site cavity. Results show that curcumin has a favorable MoleDock score due to hydrophobic and H-bonding interaction; however, competitive interaction of curcumin with natural ligands *β*-NADH to NADH-cytochrome *b*_5_ reductase require higher concentration of curcumin to downregulate the NADH-cytochrome *b*_5_ reductase activity.

Mitochondria-deficient cells (*ρ* ^0^ cells) survived through maintaining ratio of NAD/NADH and reduced coenzyme Q by upregulating the PMRS activity [31]. Intracellular redox molecule like GSH has been reported to interact and donate protons to the NADH-cytochrome *b*_5_ reductase [32]. Increased PMRS activity has been reported to associate with depletion of intracellular GSH concentration [33]. It has been reported that “redox buffering” capacity of cellular system is substantiated primarily by GSH, an indicator of the redox status of the cell [34]. Cells with low levels of GSH have been shown to be more susceptible towards irradiation and stress [35].

Here, we found that,* in vitro*, curcumin induced the redox status of erythrocytes evidenced as increased GSH concentration (Figure 6). Oral supplementation of curcumin to healthy rat also induced the GSH concentration in plasma (Figure 7(a)) as well as in erythrocytes (Figure 7(b)). It has been observed previously that oral dosing of curcumin to rats increased the hepatic cells redox status via increasing the activity of *γ*-GCS (the rate limiting step in GSH synthesis), GPx, and GST [36]. Previously we have also reported that curcumin strongly protects the GSH level in human erythrocytes against oxidative stress [19]. Our observation that curcumin supplementation reduced the PFEOP value of healthy rats indicate a decreased vulnerability of erythrocyte towards oxidative stress and hence reduced PMRS activity.

## 5. Conclusion

Results indicate that curcumin interacts with membrane bilayer to downregulate the plasma redox system activity, the effect being more pronounced at higher concentration (>10^−6^ M). In addition, curcumin also stimulates the intracellular GSH while simultaneously decreasing the oxidant capacity of plasma. Impaired redox status and altered PMRS activity are involved in various age associated diseases. The above findings provide evidence of curcumin to be an effective molecule which may be used as a supplement to combat pathologies associated with oxidative stress. Moreover the results may, in part, explain the efficacy of traditional therapeutic strategies based on the use of turmeric in prevention of age-dependent pathologies.

## Figures and Tables

**Table 1:** Amino acids showing hydrogen bond (italic) and hydrophobic interaction.

S. number	Amino acids interaction
*β*-NADH	*Arg91*, Tyr93, Ile109, Lys110, Tyr112, *Phe113*, Thr116, His117, Phe120, Gly123, Gly124, Lys125, *Met126*, *Ser127*, *Thr181*, Gln210
Curcumin (CUR)	His77, Pro92, Tyr93, Thr94, Val108, Ile109, *Lys110*, Gly179, Thr181, *Gly182*, Thr184, Pro185, Cys273, Pro275

**Table 2:** Comparative docking simulation result of selected molecule and curcumin with human NADH-cytochrome *b*_5_ reductase together with FAD, ligand from X-ray crystallized data of protein data bank (1 umk.pdb) using Molegro Virtual Docker (MVD).

S. number	Ligands	MoleDock score	H-bonding energy	Number of H-bonds
(1)	*β*-NADH	−208.235	−13.506	5
(2)	Curcumin	−141.292	−5.37453	2

## Abbreviations

PMRS: Plasma membrane redox system
CUR: Curcumin
GSH: Reduced glutathione
PFEOP: Plasma ferric equivalent oxidative potentials.
